# Draft Genome Sequences of Lactococcus lactis Strains MS22314, MS22333, MS22336, and MS22337, Isolated from Fermented Camel Milk in Ethiopia

**DOI:** 10.1128/MRA.00862-20

**Published:** 2020-11-19

**Authors:** Esben Bragason, Christina Aaby Svendsen, Mitiku Eshetu Guya, Tesfemariam Berhe, Egon Bech Hansen

**Affiliations:** a National Food Institute, Technical University of Denmark, Lyngby, Denmark; b Haramaya University, School of Animal and Range Sciences, Dire Dawa, Ethiopia; University of Southern California

## Abstract

The genome sequences of four Lactococcus lactis strains isolated from fermented camel milk were sequenced using paired-end Illumina MiSeq reads. The genome size of each strain was about 2.6 Mb, and three of the strains were annotated with *tet*(S) coding for tetracycline resistance.

## ANNOUNCEMENT

Lactococcus lactis is a well-known acidifying Gram-positive bacterium, approved with qualified presumption of safety (QPS) status by the European Food Safety Authority and used in starter cultures to make dairy products (1).

Here, we report the draft genome sequences of L. lactis strains MS22314, MS22333, MS22336, and MS22337. All strains were isolated from camel milk in Ethiopia. The new strains demonstrate superior fermentation qualities in camel milk of exponential cell growth, acidification, and decrease in redox potential, comparable to what other strains have shown in bovine milk (2). Starter cultures used for bovine-based products have shown poor fermentation results in camel milk (3, 4).

Camel milk samples (*n* = 29) were collected from several farms in the Babile area of Ethiopia and incubated at 30°C or 42°C for 48 h to stimulate fermentation. Samples with a pH of <5 after 48 h were plated and restreaked 5 times onto De Man, Rogosa, and Sharpe (MRS) agar, M17 agar containing 0.5% lactose, or Prussian blue agar, all containing 20 μg ml^−1^ natamycin for fungal inhibition (5, 6).

Single colonies from 114 isolates on one of the agar plates were chosen for 16S rRNA gene sequencing as described by Fugl et al. (2).

Based on phenotypic characterization (2), single colonies from each of four isolates, MS22314, MS22333, MS22336, and MS22337, were clean streaked at 30°C for whole-genome sequencing onto M17-lac agar plates. DNA was extracted following the manufacturer’s protocol (NORGEN milk bacterial DNA isolation kit 21550).

DNA concentrations were measured on the Qubit fluorometer using the double-stranded DNA (dsDNA) high-sensitivity (HS) assay kit (Invitrogen). Libraries for paired-end sequencing were constructed using the Nextera XT kit (Illumina, CA, USA) guide 15031942v01. The pooled Nextera XT libraries were loaded onto an Illumina MiSeq reagent cartridge using the MiSeq reagent kit v3 and 500 cycles with a standard flow cell. Both sequencing and assembly were done using default settings unless otherwise indicated. Sequencing was carried out using an Illumina MiSeq benchtop sequencer with an average read length of 210 bp, which yielded 1,868,468 to 2,187,598 reads. The coverages ranged between 130.2× and 173.8×.

The raw Illumina reads were filtered and trimmed using Assembler v1.0 (https://cge.cbs.dtu.dk/services/Assembler/) (7). The trimmed reads were assembled using Velvet v1.1.04 (8) with the standard quality control parameters included in the software. The genome statistics are reported in Table 1.

**TABLE 1:** Characteristics and accession numbers of the L. lactis strains isolated from spontaneous fermented camel milk from Ethiopia

Species and strain	Genome size (bp)	GC content (%)	No. of contigs	No. of CDS^*a*^	*N*_50_ (bp)	No. of reads	Coverage (×)	GenBank accession no.	SRA accession no.
L. lactis MS22314	2,694,284	35.0	236	2,684	66,984	1,940,308	153.9	WWDH00000000	SRR11713472
L. lactis MS22333	2,689,322	35.1	340	2,669	34,236	2,187,598	173.8	WWDI00000000	SRR11713471
L. lactis MS22336	2,692,760	35.0	273	2,690	60,629	1,667,736	130.2	WWDJ00000000	SRR11713470
L. lactis MS22337	2,659,725	35.1	233	2,674	76,645	1,868,468	147.6	WWDK00000000	SRR11713469

a CDS, coding DNA sequences.

The contigs were annotated using the NCBI Prokaryotic Genome Annotation Pipeline (PGAP) v4.11. A Swiss-Prot (9) entry (accession number Q48712) was found to have 99.8% identity to a gene coding for tetracycline resistance *tet*(S) in MS22314, MS22336, and MS22337, which should be considered when developing starter cultures for camel dairy applications.

The draft genome sequences of L. lactis strains MS22314, MS22333, MS22336, and MS22337 are valuable for future manufacturing of effective and safe starter cultures specific to the camel dairy industry.

### Data availability.

The genome sequences of MS22314, MS22333, MS22336, and MS22337 have been deposited in DDBJ/ENA/GenBank under the BioSample numbers SAMN13701540, SAMN13701541, SAMN13701542, and SAMN13701543. The raw read data have been uploaded to the NCBI Sequence Read Archive (10) and can be found at GenBank under the accession numbers listed in Table 1, together with the Illumina paired-end contigs.
